# Radial artery diameterand and age related functional maturation of the radio-cephalic arteriovenous fistula

**DOI:** 10.1186/s12882-020-01883-w

**Published:** 2020-06-22

**Authors:** Zi-ming Wan, Bo Hu, Qi-quan Lai, Xue-jing Gao, Bo Tu, Yu Zhou, Wen-bo Zhao

**Affiliations:** 1 Department of Nephrology, The First Affiliated Hospital of Chongqing Medical University, Youyi Road 1, Chongqing, 400042 China; 2 Department of Nephrology, The First Affiliated Hospital of Jinan University , Guangzhou, China; 3 Department of Ultrasonography, The First Affiliated Hospital of Chongqing Medical University, Chongqing, China; 4 Department of Nephrology, The Third Affiliated Hospital of Sun Yat-sen University, Tianhe Road NO.600, Guangzhou, 510632 China

**Keywords:** Radio-cephalic Arteriovenous fistula, Functional maturation, Age, Radial artery diameter

## Abstract

**Background:**

Previous studies have not described the relationship between reducing radial artery diameter as well as increasing age and functional maturation of the radio-cephalic arteriovenous fistula (RCAVF) and no data identify these as linear relationship. The objective of this study was to perform trend analysis to assess these aspects.

**Methods:**

Our retrospective cohort study enrolled and analyzed 353 follow-up cases that underwent first AVF creation. The artery and vein sizes were measured by ultrasound. We performed follow-up, a minimum of 3 months after surgery. Multivariable logistic regression analysis was used to identify independent risk factors inmaturation. Participant age was categorized into four groups (age ≤ 29, 30–49, 50–69, and 70–90 years). Radial artery diameter was categorized into four groups (≤ 1.9, >1.9 and ≤ 2.1, >2.1 and ≤ 2.4, >2.4 mm) according to median and interquartile ranges. We adjusted for confounders in four logistic models, and primary analyses were based on building ordered category models and tested ***P*** values for trends to estimate the relationship of radial artery diameter and each 20-year increase in age with risk of maturation.

**Results:**

The mature RCAVF group included 301 cases, and the immature group included 52 cases. Radial artery diameter, age, and diabetes were independent risk factors of maturation. Odds ratios (ORs) associated with maturation reduced with increasing age, while ORs increased with increasing radial artery diameter. ***P*** values for trends(**<0.05**) were observed in all four models. A reduction in radial artery diameter and higher age were significantly associated with a higher incidence of immaturity after adjusting the multivariate models. The risks of immaturation were increased by more than 1.54 fold for each 20-year increase and increased by more than 1.34 fold for the smaller radial artery diameter group.

**Conclusion:**

Our findings suggest that a significantly higher immaturity risk of RCAVF was associated with increasing age and a reduction in radial artery diameter. Our study identified a linear exposure-response relationship of age and radial artery diameter with immaturity incident. A careful selection of patients will be helpful in improving AVF functional maturation.

## Background

Vascular access is the “life-line” for hemodialysis in patients. Functional maturation of the arteriovenous fistula (AVF) is vital for hemodialysis treatment. Autogenous AVFs are the first choice for vascular access for hemodialysis in patients [1]. AVF is the best vascular access for hemodialysis in patients; it allows better follow-up; has better outcomes [2, 3].

Overall, 28 to 53% of AVFs have been reported to fail to develop sufficiently for dialysis [4]. AVFs are associated with high non-maturation rates [5]. Multiple factors, including age and vessel diameter, are involved in functional maturation [6]. Older people havea high risk for diabetes and peripheral vascular disease. The literature reports on influence of age on access maturation are conflicting. Some studies have reported no difference in AVF maturation with advanced age [7–9] while other studies have reached contrasting conclusions, showing that elderly individuals with radio-cephalic AVFs (RCAVFs) had lower maturation [10–14]. Therefore, the optimal vascular access in the elderly for renal replacement therapy (RRT) remains controversial [15].

A diameter of the radial artery in the range of 1.5 to > 2 mm with regard to maturation and primary patency is highly advocated [16]; however, the relationship between artery diameter change and maturation rate is unclear.

There are no data to identify whether there is a linear relationship of increasing age and reducing radial artery diameter with functional maturation The objective of the present study was to perform a trend analysis of increasing age and reducing radial artery diameter with primary functional maturation using a retrospective cohort, which can be helpful in medical decision-making for dialysis patients.

## Methods

### Selection of patients

**Fig. 1:**
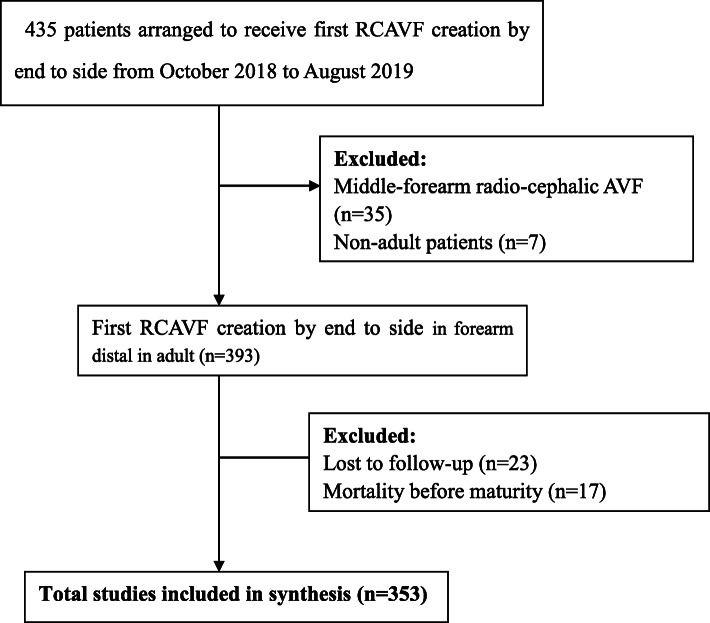
Flow chart of study participants

Our study population was derived from a retrospective cohort with end-stage renal disease (ESRD) and who underwent hemodialysis from 10/1/2018 to 8/30/2019 at the Department of Nephrology, the primary affiliated hospital of Chongqing medical university. The patients were followed monthly at the outpatient clinic or in-person by telephone as per patient’s preference or convenience. A total of 435 adult patients were planned to receive RCAVF creation under local anesthesia in this study. Our study excluded patients who had undergone middle-forearm radio-cephalic AVF creation and patients less than 18 years of age (*n* = 42). We confirmed that 393 patients had received first RCAVF creation by end-to-side anastomosis. We followed patients after surgery until death, loss to follow-up, or failure to maturewith a minimum follow-up of 4 months. We excluded patients who died before maturity and those who were lost to follow-up (*n* = 40). Ultimately, our study enrolled and analyzed 353 follow-up cases. The selection of study participants is described in Fig. 1. Patients’ demographics included age, sex, body mass index, and smoking history. Diabetes was included in the comorbidities analysis. Longitudinal measures of laboratory parameters (e.g. hemoglobin, serum calcium, serum phosphorus, albumin, coagulation factors, and blood lipids) were collected, and functional maturation was assessed. Ultrasound examination was performed before surgery. All ultrasound examinations were carried out by the same sonographer. Multiple sites evaluation of fistulae was carried out from the brachial artery to distal radial artery, and from distal cephalic vein to upper arm cephalic vein. After bundling arm for more than 1 min, cephalic vein diameters were measured. Two different cross-sectional vascular diameters of cephalic vein and radial artery at surgical sites were measured at the vessel wall interface and an average of the two diameters was recorded.

All vascular access types were created by two experienced nephrologists. AVF was created under local anesthesia by direct anastomosis between the end of the vein and the side of the artery. The threshold diameter of abandoning an RCAVF creation was as follows: radial artery < 1.5 mm or cephalic vein < 2.0 mm.

### Definition of functional maturation

The functional maturation of RCAVFs was an outcome of the study. Functional maturation was defined as successful 2-needle annulations during hemodialysis treatment at 4–6 weeks after surgery and a blood flow rate over 200 ml/min for at least six consecutive hemodialysis sessions. An immature fistula was defined as one that was difficult to cannulate, one that was never mature enough to be used, and/or failure to generate a sufficient blood flow rate [17].

### Data analysis and statistics

The study cohort was categorized into four groups based on age:≤29, 30–49, 50–69, and 70–90 years according to 20-year increments. Radial artery diameter was categorized into four groups (≤ 1.9, >1.9 and ≤ 2.1, >2.1 and ≤ 2.4, and >2.4 mm) according to the median and interquartile ranges. We adjusted for several confounders in our logistic models. Model1 was adjusted for age or radial artery diameter. In model 2, we further adjusted for various covariates, including sex, smoking status, body mass index (BMI), and diabetes. In model 3, based on model 2, we further adjusted for cephalic vein diameter and surgical sites (left side or right side). In model 4, we further adjusted for laboratory measures (e.g. hemoglobin, serum calcium, serum phosphorus, albumin, coagulation factors, and blood lipids). Primary analyses were based on tests for trends according to the age or radial artery diameter categories.

All data were analyzed using SPSS 25.0 software (IBM Corp., Armonk, NY, USA). In the baseline characteristics table, all the continuous variables with non-normal distribution are expressed as medians and interquartile ranges (IQR) and were tested by the Kolmogorov-Smirnov test. Categorical variables are shown as the percentage of patients per group. All the categorical variables were analyzed with the Chi-squared test. The univariate and multivariable logistic regression models were used to identify risk factors of functional maturation. Logistic regression estimated the odds ratios (ORs) and 95% confidence intervals (CIs). ***P*** values for trends were used to estimate the trend of relationship of each 20-year increase in age and radial artery diameter with maturity risk. A ***P*** value < 0.05 was defined as statistically significant.

## Results

### Characteristics of participants

The baseline characteristics of the 353 follow-up RCAVF cases and clinical data of the study participants are summarized in Table 1**.** The mature RCAVF group included 301 cases, and the immature RCAVF group included 52 cases. The participants in the immature RCAVF group were significantly older and their wrist radial artery diameter was significantly smaller than those in the mature RCAVF group. Number of patients with diabetes was higher in the mature RCAVF group. However, between-group differences in sex, smoking history, BMI, surgical sites (left side or right side), cephalic vein diameter, and laboratory measures (e.g. hemoglobin, serum calcium, serum phosphorus, albumin, coagulation factors, and blood lipids) were not significant. (Table 1).

**Table 1:** Summary of the baseline characteristics of wrist RCAVFs

Variable		mature Wrist RCAVF group	Immature Wrist RCAVF group	z/χ2	*P*
		(*n* = 301)	(*n* = 52)		
Gender	male(%)	122 (40.50)	22 (43.20)	5.326	0.021
	female(%)	179 (59.5)	30 (57.70)		
Age (years), Median (IQR)		57.00 (46.00, 67.00)	61.50 (52.00, 74.00)	−2.611	0.009
Diabetes (%)		111 (36.90)	27 (51.90)	4.216	0.040
Smoking history (%)		129 (42.90)	19 (36.50)	0.727	0.394
BMI/(kg/m^2^),Median (IQR)		22.99 (20.76, 25.66)	21.87 (19.92, 24.20)	−1.734	0.083
Left side (%)		253 (84.10)	41 (78.80)	0.864	0.353
Wrist radial artery diameter (mm),Median (IQR)		2.10 (1.90, 2.50)	2.00 (1.80, 2.20)	−2.812	0.005
Wrist cephalic vein diameter (mm),Median (IQR)		2.50 (2.20, 2.80)	2.30 (2.10, 2.70)	−1.694	0.090
HGB/(g/L), Median (IQR)		86.00 (75.00, 98.00)	83.50 (74.00, 103.75)	−0.222	0.824
Albumin/ (g/L), Median (IQR)		36.00 (32.00, 39.00)	35.00 (32.00, 37.38)	−0.599	0.549
Ca/(mmol/L), Median (IQR)		2.04 (1.89, 2.18)	2.07 (1.92, 2.21)	−1.081	0.280
P/(mmol/L), Median (IQR)		1.78 (1.46, 2.20)	1.73 (1.29, 2.45)	−1.651	0.099
PTH/(pg/ml), Median (IQR)		286.30 (211.20, 387.40)	270.98 (192.10, 406.80)	−0.541	0.589
TC/(mmol/L), Median (IQR)		3.89 (3.37,4.35)	3.93 (3.25, 4.92)	−0.265	0.791
TG/(mmol/L), Median (IQR)		1.37 (1.06, 1.80)	1.48 (1.05, 2.48)	−1.343	0.179
LDL-C/(mmol/L), Median (IQR)		2.25 (1.80, 2.63)	1.96 (1.66, 3.06)	−0.951	0.341
HDL-C/(mmol/L), Median (IQR)		1.09 (0.88, 1.28)	1.10 (0.85, 1.30)	−0.096	0.923
FBG/ (s), Median (IQR)		4.30 (3.67, 5.19)	4.04 (3.12, 4.84)	−1.887	0.059

*BMI* Body mass index; *HGB* Hemoglobin; *Ca* Serum calcium; *P* Serum phosphorus; *PTH* parathyroidhormone; *TC* Total Cholesterol; *TG* Triglyceride; *HDL-C* High-density Lipoprotein; *LDL-C* Low Density Lipoprotein; *PT* Prothrombin time; *APTT* Activated partial thromboplastin time; *INR* International normalized ratio; *FBG* Fibrinogen

### Multivariate logistic regression analysis and trend analysis of maturation outcome in multiple models

Results of the multivariate logistic regression analysis for risk factors of maturation are shown in Table 2**.** Age, radial artery diameter, and diabetes were independent predictors for maturation. Increasing age and reducing radial artery diameter as continuous variables suggested elevated immaturity risk. In order to compare increasing age and reducing radial artery diameter in relation to reducing primary functional maturation, we used a trend analysis of maturation for different age (≤ 29, 30–49, 50–69, and 70–90 years) and radial artery diameter(≤ 1.9, >1.9 and ≤ 2.1, >2.1 and ≤ 2.4, and >2.4 mm) categories as components in multiple models. The ORs associated with maturation reduced with increasing age and increased with increasing radial artery diameter. ***P*** values for trends(**<0.05**) were all observed in all four models (Table 3**,** Table 4). In the age-adjusted models, higher age was significantly associated with an increased risk of immaturity (*P* < 0.05). In the radial artery diameter adjusted models, increasing radial artery diameter was significantly associated with a reduced risk of immaturity (*P* < 0.05). After multivariate adjustment, we observed similar results.

**Table 2:** The multivariate regression analysis for maturation outcomes of RCAVFs

Variable	B	SE	Wald	df	Sig.	Exp(B)	95.0% CI for Exp(B)	
							Lower	Upper
Age	−0.029	0.013	4.994	1	0.025	0.971	0.946	0.996
Diabetes	−0.939	0.391	5.753	1	0.016	0.391	0.182	0.842
Wrist radial artery diameter	1.045	0.458	5.201	1	0.023	2.843	1.158	6.980

**Table 3:** Trend analysis of maturation outcomes for different age levels components in multiple models

Variable					***P*** to trend
	20–29	30–49	50–69	70–90	
Age					
Median (y)	24.50	44.00	60.00	74.50	
Cases/person	1/24	10/83	22/164	19/82	
Model 1, OR(95%CL)	1.00(Ref)	0.317 (0.039, 2.614)	0.281 (0.036, 2.184)	0.144 (0.018, 1.139)	0.014
***P*** Value		0.286	0.225	0.066	
Model 2, OR(95%CL)	1.00(Ref)	0.341 (0.040, 2.894)	0.369 (0.045, 2.989)	0.175 (0.021, 1.443)	0.041
***P*** Value		0.324	0.350	0.105	
Model 3, OR(95%CL)	1.00(Ref)	0.276 (0.031, 2.431)	0.316 (0.038, 2.656)	0.135 (0.016, 1.172)	0.029
***P*** Value		0.246	0.289	0.069	
Model 4, OR(95%CL)	1.00(Ref)	0.236 (0.026, 2.171)	0.313 (0.036, 2.723)	0.126 (0.014, 1.133)	0.039
***P*** Value		0.202	0.293	0.065	

*OR* Odds rations; *95% CL* 95% Confidence interval; Model 1: unadjusted relevant factors; Model 2: Univariate model plus gender,

*BMI* Smoking history, diabetes; Model 3: Model 2 plus wrist radial artery diameter, wrist cephalic vein diameter, left side or right

side; Model 4: Model 3 plus HGB, albumin, Ca, P, PTH, TC, TG, LDL-C, HDL-C, FBG

**Table 4:** Trend analysis of immaturation outcomes for different radial artery diameter components in multiple models

Variable	Radial artery diameter (mm)				***P*** to trend
	≤1.9	>1.9 and ≤ 2.1	>2.1and ≤2.4	>2.4	
Diameter					
Median (y)	1.80	2.10	2.30	2.70	
Cases/person	22/107	14/83	9/76	7/87	
Model 1, OR(95%CL)	1.00(Ref)	0.338 (0.137, 0.835)	0.431 (0.165, 1.129)	0.651 (0.230, 1.842)	0.011
*P* Value		0.059	0.087	0.419	
Model 2, OR(95%CL)	1.00(Ref)	1.251 (0.573, 2.732)	1.831 (0.768, 4.363)	2.841 (1.085, 7.441)	0.023
*P* Value		0.575	0.172	0.034	
Model 3, OR(95%CL)	1.00(Ref)	1.231 (0.563, 2.692)	1.820 (0.763, 4.339	2.765 (1.048, 7.295)	0.027
*P* Value		0.603	0.177	0.040	
Model 4, OR(95%CL)	1.00(Ref)	1.181 (0.531, 2.625)	1.592 (0.659, 3.848)	2.638 (0.977, 7.127)	0.045
*P* Value		0.683	0.302	0.056	

*OR* Odds rations; *95% CL* 95% Confidence interval; Model 1: unadjusted relevant factors; Model 2: Univariate model plus age,

gender, BMI, smoking history, diabetes; Model 3: Model 2 plus wrist cephalic vein diameter, left side or right side;

Model 4: Model 3 plus HGB, albumin, Ca, P, PTH, TC, TG, LDL-C, HDL-C, FBG

### Multiple logistic regression models to analyze immaturity risk

Age was used as a categorical variable, and every 20-year increase in age was significantly associated with a higher OR (OR > 1.543) for the incidence of immaturity after multivariate adjustment (Table 5). A smaller diameter of the artery was significantly associated with a higher OR (OR > 1.349) for immaturity, after multivariate adjustment, when the radial artery diameter was considered as a categorical variable (Table 6).

**Table 5:** Multiple models of logistic regression to analyze immaturity risk for different age levels

Variable	B	SE	Wald	df	Sig.	Exp(B)	95.0% CI for Exp(B)	
							Lower	Upper
Age								
Model 1	0.482	0.194	6.188	1	0.013	1.620	1.108	2.368
Model 2	0.434	0.208	4.343	1	0.037	1.543	1.026	2.320
Model 3	0.478	0.214	4.980	1	0.026	1.614	1.060	2.456
Model 4	0.460	0.218	4.446	1	0.035	1.584	1.033	2.430

Model 1: Unadjusted relevant factors; Model 2: Univariate model plus gender, BMI, smoking history, diabetes; Model 3: Model 2 plus wrist radial artery diameter, wrist cephalic vein diameter, left side or right side; Model 4: Model 3 plus HGB, albumin, Ca, P, PTH, TC, TG, LDL-C, HDL-C, FBG

**Table 6:** Multiple models of logistic regression to analyze immaturity risk for different radial artery diameter levels

Variable	B	SE	Wald	df	Sig.	Exp(B)	95.0% CI for Exp(B)	
							Lower	Upper
Wrist radial artery diameter								
Model 1	0.354	0.138	6.569	1	0.010	1.425	1.087	1.868
Model 2	0.337	0.147	5.284	1	0.022	1.401	1.051	1.869
Model 3	0.330	0.148	5.000	1	0.025	1.391	1.042	1.858
Model 4	0.299	0.150	3.997	1	0.046	1.349	1.006	1.808

Model 1: Unadjusted relevant factors; Model 2: Univariate model plus age, gender, BMI, smoking history, diabetes; Model 3: Model 2 plus wrist cephalic vein diameter, left side or right side; Model 4: Model 3 plus HGB, albumin, Ca, P, PTH, TC, TG, LDL-C, HDL-C, FBG

## Discussion

In this prospective cohort study, we found a significantly higher risk of immaturity of RCAVF associated with increasing age and reducing diameter of the radial artery. Our study identified an almost linear exposure-response relationship of age and radial artery diameter with incident immaturity. Older patients showed a greater prevalence and risk of immaturity. Each 20-year increase in age was significantly associated with an increase of more than 54% in immaturity risk after multivariate adjustment. A smaller artery diameter was related to a higher immaturity rate. Compared with the highest quartile of artery diameter, participants in the lower quartile had an increased risk of incident immaturity (by more than 34%).

As populations are rapidly ageing worldwide, associated health issues have increased. The age of individuals requiring dialysisis is increasing. The elderly population requiring RRT is growing worldwide, comprising no less than 25 to 30% in most ESRD registration systems [18, 19]. In Catalan, more than half of the patients planned to start dialysis are over 60 years of age [20]. In the United States, from 1996 until 2003, the dialysis initiation rate among patients aged > 65 years has increased by nearly 10% annually, with an overall increase of 57% [21]. In Canada, the proportion of patients aged 75 and older starting dialysis has increased by 74% between 1990 and 2001 [21].

Autogenous AVFs are recommended as the first choice for hemodialysis vascular access [22]. Previous studies reported that elderly patients with RCAVFs had lower maturation [10–14]. A meta-analysis showed that elderly patients had a 50% increased RCAVF failure risk when compared with non-elderly patients [10]. Older patients with higher intima-media width and arterial rigidity showed increased vessel narrowing and loss of vascular elasticity [23]. The prevalence of diabetes and peripheral vascular disease increases in older individuals, and when compared with subjects without chronic kidney diseases, dialysis patients had a higher incidence of diabetes and peripheral vascular disease [24] that was in turn associated with increasing immaturity. Previous several comparative studies [25–27] supported pre-operative ultrasound scanning. European Best Practice Guidelines suggest its routine use (level II evidence) [21]. Several meta-analysis [28–30] including only one of the few randomized clinical trials (RCTs) appeared contradicting results. Georgiadis et al. study [29] found pre-operative ultrasound mapping to avoid unnecessary surgical explorations and significantly reduces the immediate AVF failure rate. We considered that the pre-operative clinical examination and routine ultrasound mapping should always be performed before AVF creation. If the patient has obvious intra-arterial lesions, such as significant intimal hyperplasia, severe calcification, and vascular malformations, which cannot meet the requirements of the surgical diameter, we will not perform the surgery.

The optimal vascular access for RRT in the elderly is controversial [15]. Elbow brachiocephalic fistulas and arteriovenous grafts (AVGs) may be important alternatives in elderly patients [10]. Our study highlights a positive correlation between increasing age and the incidence of immaturity. Therefore, a careful evaluation of clinical characteristics and a clinical scoring system to establish what is essential for the increasingly ageing dialysis patient population are imperative for accurate medical decision-making.

In practice, the diameters of the radial artery and cephalic vein are evaluated by preoperative duplex ultrasound. In our study, all ultrasound examinations were carried out by the same sonographer. Niek et al. study [31] indicated that the arterial and venous diameter measurements showed excellent intra- and inter-observer agreement and duplex ultrasonography was a reliable imaging modality to support AVF surgery planning. There are no definite criteria for determining a suitable artery for AVF. Radial artery diameter within a range of 1.5 to > 2 mm is usually selected. However, there are no data concerning the relationship of radial artery diameter with functional maturation. Our study shows that there is a linear relationship between radial artery diameter and incident immaturity. This may be beneficial in operational risk assessments and medical decision-making.

## Conclusions

Our findings suggest that the risks of immaturity of RCAVF were linearly associated with increasing age and reducing radial artery diameter. Each 20-year increase in age (risk increased by more than 54%) and a smaller radial artery diameter (risk increased by more than34%) were associated with a higher incidence of immaturity. A careful selection of patients in different age groups who are suitable for AVFs may be helpful to improve functional maturation. Some limitations of our study must be acknowledged. Firstly, this study had a low number of patients in some groups and further studies should be performed with larger sample sizes. Secondly, many potential factors should be considered that were not evaluated in our study (e.g. peripheral vascular disease).

## Supplementary information

**Additional file 1.**

**Additional file 2.**

## Abbreviations

RCAVF: Radio-cephalic arteriovenous fistula
AVF: Arteriovenous fistula;
ORs: Odds ratios
RRT: Renal replacement therapy
ESRD: End-stage renal disease
IQR: Interquartile ranges
BMI: Body mass index
AVGs: Arteriovenous grafts
