# Supplementary material for: Radial artery diameterand and age related functional maturation of the radio-cephalic arteriovenous fistula
Source: BMC Nephrol. 2020 Jun 22;21:234. doi: 10.1186/s12882-020-01883-w (PMC7310035; doi:10.1186/s12882-020-01883-w)
Supplement: Supplementary file 2 — Additional file 2. [file 12882_2020_1883_MOESM2_ESM.docx]

**BMC Nephrology	BNEP-D-20-00183	22 May 2020**

**Radial artery diameter and age related** **functional** **maturation of the radio-cephalic arteriovenous fistula**

Zi-ming Wan; Bo Hu; Qi-quan Lai; Xue-jing Gao; Bo Tu; Yu Zhou; Wenbo Zhao

Dear Dr. Robin L. Cassady-Cain:

Thank you for your letter and for the reviewers’ comments concerning our manuscript, entitled “Radial artery diameter and age related functional maturation of the radio-cephalic arteriovenous fistula” (**BNEP-D-20-00183**). The comments are all valuable and very helpful for revising and improving our paper, as well as the important guiding significance to our researches. We have studied comments carefully again and have made correction again which we hope meet with approval. Revised portion are marked in blue in the responds. The main corrections in the paper and the responds to the reviewer’s comments are as flowing:

**Reviewer reports:**
**Reviewer 1:** The authors significantly improved the manuscript.
The conclusion is limited by the very nature of their study but the statistical finding is clear and interesting for the community.

**Answer:** Thanks for your hard work at the COVID-19 pandemic period.

**Reviewer 2:** Most of the reviewers comments are answered. there remain still some issues:

1. You have not adequately responded on the role of intra and inter-observer variability of the ultrasound measurements. At least pay some attention to this issue in your discussion.

**Answer:** Per your suggestion, we have revised the manuscript about the role of intra and inter-observer variability of the ultrasound measurements in discussion.

“ In practice, the diameters of the radial artery and cephalic vein are evaluated by preoperative duplex ultrasound. In our study, all ultrasound examinations were carried out by the same sonographer. Niek et al. study[31] indicated that the arterial and venous diameter measurements showed excellent intra- and inter-observer agreement and duplex ultrasonography was a reliable imaging modality to support AVF surgery planning.”

**References**

31. Zonnebeld N, Maas T, Huberts W, van Loon MM, Delhaas T, Tordoir J. Pre-operative Duplex Ultrasonography in Arteriovenous Fistula Creation: Intra- and Inter-observer Agreement. Eur J Vasc Endovasc Surg. 2017;54:613-9.

2. page 11: Several meta-analysis [28-30] including only one of the few randomized clinical trials (RCTs) appeared contradicting results and usually selected. However, there are no data concerning the relationship of radial artery diameter with functional maturation. Our study shows that there is a linear investigating, Georgiadis et al. study found pre-operative ultrasound mapping to avoid negative surgical explorations and significantly reduces the immediate AVF failure rate. "
   This sentence is difficult to understand. Please improve the language.
   **Answer:** The comment is of significant importance. We have re-written this part, and adjusted the logical relationship of sentences, according to the Reviewer’s suggestion.

**Editorial Policies**

Authors who do not wish to share their data must confirm this under this sub-heading and also provide their reasons.
For further guidance on how to format this section, please refer to BioMed Central's editorial policies page (see links below).“ Declarations; Ethics approval and consent to participate; Consent to publish; Availability of data and materials; Competing interests; Funding; Authors' Contributions; Acknowledgements”
**Answer:** Considering **Editorial Policies，**we have revised the manuscript

We tried our best to improve the manuscript and made some changes in the manuscript. These changes will not influence the content and framework of the paper. And here we did not list the changes but marked in revised paper.

We appreciate for Editors and Reviewers’ warm work earnestly, and hope that the correction will meet with approval.

Thank you very much again for your hard work at the COVID-19 pandemic period.

Thank you for your comments and suggestions.

Wen-bo Zhao

Corresponding authors: Wenbo Zhao

E-mail: bobo800101@foxmail.com
